# On Critical Buckling Loads of Columns under End Load Dependent on Direction

**DOI:** 10.1155/2014/531438

**Published:** 2014-10-29

**Authors:** Musa Başbük, Aytekin Eryılmaz, M. Tarık Atay

**Affiliations:** ^1^Department of Mathematics, Nevşehir Hacı Bektaş Veli University, 50300 Nevşehir, Turkey; ^2^Department of Mechanical Engineering, Abdullah Gül University, 38039 Kayseri, Turkey

## Abstract

Most of the phenomena of various fields of applied sciences are nonlinear problems. Recently, various types of analytical approximate solution techniques were introduced and successfully applied to the nonlinear differential equations. One of the aforementioned techniques is the Homotopy analysis method (HAM). In this study, we applied HAM to find critical buckling load of a column under end load dependent on direction. We obtained the critical buckling loads and compared them with the exact analytic solutions in the literature.

## 1. Introduction

Nonlinear differential equations arise in a wide range of scientific studies from physics to biology, from engineering to economics. However it is not possible to find an exact analytical solution for the nonlinear equations every time. Analytical approximate solution techniques such as perturbation and nonperturbative techniques have been used to solve these nonlinear equations in recent years. These techniques have been widely applied in many fields of engineering and science. Neither perturbation techniques nor nonperturbation techniques ensure the convergence of solution series and adjust or control the convergence region and rate of approximation series.

On the other hand an analytic approach, the homotopy analysis method (HAM) which is proposed by Liao, provides a convenient way to adjust and control the convergence region and the rate of approximation series by the auxiliary parameter *ħ* and auxiliary function *H*(*t*) [1, 2]. HAM has been applied successfully to obtain the series solution of various types of linear and nonlinear differential equations such as the viscous flows of non-Newtonian fluids [3–13], the KdV-type equations [14–16], nanoboundary layer flows [17], nonlinear heat transfer [18, 19], finance problems [20, 21], Riemann problems related to nonlinear shallow water equations [22], projectile motion [23], Glauert-jet flow [24], nonlinear water waves [25], ground water flows [26], Burgers-Huxley equation [27], time-dependent Emden-Fowler type equations [28], differential difference equation [29], difference equation [30], Laplace equation with Dirichlet and Neumann boundary conditions [31], and thermal-hydraulic networks [32].

One of the fields that nonlinear differential equations arise is the stability analysis of columns in mechanical engineering. Many researchers applied analytical approximate solution techniques to the stability analysis of various types of columns with different end conditions. Atay and Coşkun investigated the elastic stability of a homogenous and nonhomogenous Euler beam [33–39]. Pinarbasi investigated the buckling analysis of nonuniform columns with elastic end restraints [40]. Huang and Luo determined critical buckling loads of beams with arbitrarily axial inhomogeneity [41]. Recently, Yuan and Wang [42] solved the postbuckling differential equations of extensible beam-columns with six different cases. Eryılmaz and Atay investigated the buckling loads of Euler column with a continuous elastic restraint by using HAM [43].

In this study we apply HAM to find the critical buckling load of a column under end load dependent on direction.

## 2. Column under End Load Dependent on Direction

**Figure 1:**
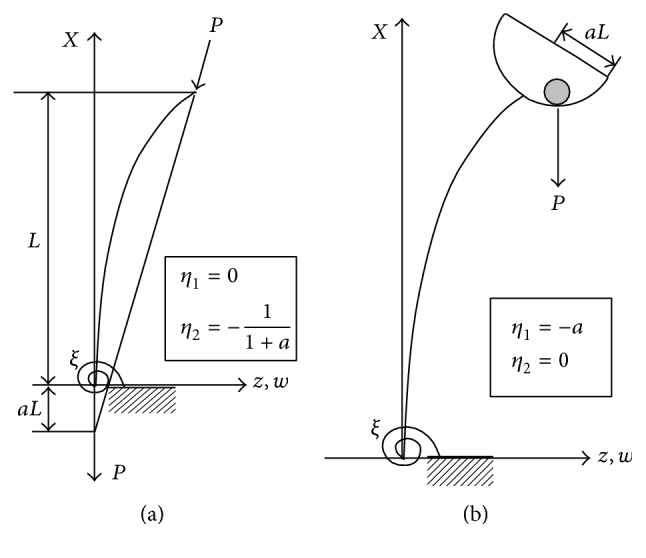
Buckling of various types of columns [45].

Consider a fixed-free, uniform homogeneous column of flexural rigidity EI, length *L* which is subjected to a load *P* that is dependent on the deflection and slope of the free end of the buckled column as shown in Figure 1 [44].

The governing buckling equation is given by [45]

(1) $$\begin{array}{c} \frac{d^{4}w}{dx^{4}}+\alpha \frac{d^{2}w}{dx^{2}}=0,\;\alpha =\frac{PL^{2}}{\text{El}}, \end{array}$$

subject to the boundary conditions:

(2) $$\begin{array}{c} w\left(0\right)=0,\\ {\left[\xi_{0}\frac{dw}{dx}-\;\frac{d^{2}w}{dx^{2}}\right]|}_{x=0}=0,\\ {\left[\alpha \eta_{1}\frac{dw}{dx}+\frac{d^{2}w}{dx^{2}}\right]|}_{x=1}=0,\\ \alpha \eta_{2}w\left(1\right)+{\left[\;\frac{d^{3}w}{dx^{3}}+\alpha \frac{dw}{dx}\right]|}_{x=1}=0, \end{array}$$

where *η*_1_ and *η*_2_ are nondimensional parameters defined in Figure 1. The general solution of (1) is

(3) $$\begin{array}{c} w=c_{1}\sin\sqrt{\alpha }x+c_{2}\cos\sqrt{\alpha }x+c_{3}x+c_{4}. \end{array}$$

Substituting the general solution into the aforementioned boundary conditions, the stability criteria take the following form [45]:

(4) 2+[1αη1+1ξ+1η1η2ξ+1η1ξ−1η2−1]αsinα   +[2−α(1η2+1)(1αη1+1ξ)]cos⁡α=0.

The stability criteria for the columns in Figure 1 are given in Table 1.

## 3. Basic Idea of Homotopy Analysis Method (HAM)

Liao introduced the homotopy analysis method (HAM) in [1, 2]. To demonstrate the homotopy analysis method, let us consider the following differential equation:

(5) $$\begin{array}{c} N\left[w\left(x\right)\right]=0, \end{array}$$

where *N* is a nonlinear operator, *x* denotes the independent variable, and *w*(*x*) is an unknown function. Liao [2] constructs the so-called zero order deformation equation as follows:

(6) $$\begin{array}{c} \left(1-q\right)L\left[\phi \left(x;q\right)-w_{0}\left(x\right)\right]=q\hbar H\left(x\right)N\left[\phi \left(x;q\right)\right], \end{array}$$

where *q* ∈ [0,1] is the embedding parameter, *ħ* is a nonzero auxiliary linear parameter, *H*(*x*) is nonzero auxiliary function, *w*_0_(*x*) is the initial guess of *w*(*x*), *L* is an auxiliary linear operator, and *ϕ*(*x*; *q*) is an unknown function. As *q* increases from 0 to 1, *ϕ*(*x*; *q*) varies from the initial guess *w*_0_(*x*) to the exact solution *w*(*x*). By expanding *ϕ*(*x*; *q*) in a Taylor's series with respect to *q*, one has

(7) $$\begin{array}{c} \phi \left(x;q\right)=w_{0}\left(x\right)+\overset{\infty }{\underset{m=1}{\sum }}w_{m}\left(x\right)q^{m}, \end{array}$$

where

(8) $$\begin{array}{c} w_{m}\left(x\right)={\frac{1}{m!}\frac{\partial^{m}N[\phi (x;q)]}{\partial q^{m}}|}_{q=0}. \end{array}$$

If the initial guess, auxiliary linear operator, auxiliary parameter, and auxiliary function are properly chosen the series (8) converges at *q* = 1; then we have

(9) $$\begin{array}{c} w\left(x\right)=w_{0}\left(x\right)+\overset{\infty }{\underset{m=1}{\sum }}w_{m}\left(x\right). \end{array}$$

Let us define the vector

(10) $$\begin{array}{c} {\vec{w}}_{m}\left(x\right)=\left\{w_{1}\left(x\right),w_{2}\left(x\right),\ldots ,w_{n}\left(x\right)\right\}. \end{array}$$

Differentiating equation (6) *m*-times with respect to *q* and then setting *q* = 0 and finally dividing by *m*!, Liao has the so-called *m*th order deformation equation:

(11) $$\begin{array}{c} L\left[w_{m}\left(x\right)-\chi_{m}w_{m-1}\left(x\right)\right]=\hbar H\left(x\right)R_{m}\left[{\vec{w}}_{m-1}\left(x\right)\right], \end{array}$$

where

(12) $$\begin{array}{c} R_{m}\left[{\vec{w}}_{m-1}\left(x\right)\right]={\frac{1}{\left(m-1\right)!}\frac{\partial^{m-1}N\left[\phi \left(x;q\right)\right]}{\partial q^{m-1}}|}_{q=0}, \end{array}$$

(13) $$\begin{array}{c} \chi_{m}=\{\begin{array}{cc} 0, & m\leq 1,\\ 1, & m>1. \end{array} \end{array}$$

In order to obey both of* the rule of solution expression* and* the rule of the coefficient ergodicity* [2], the corresponding auxiliary function is determined by *H*(*x*) = 1. For any given operator *N*, the term $R_{m}[{\vec{w}}_{m-1}(x)]$ can be easily expressed by (12). So we can obtain *w*_1_(*x*), *w*_2_(*x*),… by means of solving the linear high order deformation equation (11). The *m*th order approximation of *W*(*x*) is given by

(14) $$\begin{array}{c} w\left(x\right)≅W\left(x\right)=\overset{n}{\underset{m=0}{\sum }}w_{m}\left(x\right). \end{array}$$

The approximate solution consists of *ħ*, which is a cornerstone of the HAM in determining convergence of series solution rapidly. We may adjust and control the convergence region and rate of the solution series (14) by means of the auxiliary parameter *ħ*. To obtain valid region of *ħ* we first plot the so-called *ħ*-curves of *W*(*x*, *ħ*). The valid region of *ħ* is the interval, which corresponds to the line segments nearly parallel to the horizontal axis.

**Theorem 1 (Convergence Theorem [2]).**

As long as the series (9) converges to *w*(*x*), where *w*_*m*_(*x*) is governed by the high order deformation equation (11) under the definitions (12) and (13), it must be the exact solution of (1) subject to the boundary conditions (2).

For the proof see [2].

## 4. HAM Formulation of the Problem

To solve (1) by means of homotopy analysis method, we define the nonlinear operator *N*[*ϕ*(*x*; *q*)] and the auxiliary linear operator *L* as follows:

(15) $$\begin{array}{c} N\left[\phi \left(x;q\right)\right]=\phi^{\left(\text{ıv}\right)}\left(x;q\right)+\alpha \phi^{\prime \prime }\left(x;q\right),\\ L\left[\phi \left(x;q\right)\right]=\phi^{\left(\text{ıv}\right)}\left(x;q\right). \end{array}$$

Using the embedding parameter *q* ∈ [0,1], we construct a family of equations:

(16) $$\begin{array}{c} \left(1-q\right)L\left[\phi \left(x;q\right)-w_{0}\left(x\right)\right]=q\hbar H\left(x\right)N\left[\phi \left(x;q\right)\right]. \end{array}$$

The high order deformation equation is as follows:

(17) $$\begin{array}{c} L\left[w_{m}\left(x\right)-\chi_{m}w_{0}\left(x\right)\right]=\hbar H\left(x\right)R_{m}\left[{\vec{w}}_{m-1}\left(x\right)\right], \end{array}$$

where

(18) $$\begin{array}{c} R_{m}\left[{\vec{w}}_{m-1}\left(x\right)\right]=w_{m-1}^{\left(\text{ıv}\right)}\left(x\right)+\alpha w_{m-1}^{\prime \prime }\left(x\right). \end{array}$$

By using (17) and (18), choosing *H*(*x*) = 1, the high order deformation equation (17) yields the equation

(19) wm(x) =χmwm−1(x)+ħ  ×∫0x∫0τ∫0ζ∫0ψ[wm−1(ıv)(ξ)+αwm−1′′(ξ)]dξ dψ dζ dτ.

Starting with an initial approximation *w*_0_(*x*), we successively obtain *w*_*i*_(*x*), *i* = 1, 2, 3,…, by (19). The solution is of the form

(20) $$\begin{array}{c} w\left(x\right)=w_{0}\left(x\right)+\overset{\infty }{\underset{m=1}{\sum }}w_{m}\left(x\right). \end{array}$$

Since the governing equation (1) is a fourth order differential equation we choose the initial approximation as *w*_0_(*x*) = *ax* ^3^ + *bx* ^2^ + *cx* + *d* a polynomial of third degree with four unknown coefficients *a*, *b*, *c*, *d*. Then we obtained *w*_*i*_(*x*), *i* = 1,2, 3,…, by using the *m*th order deformation equation (19) as follows:

(21) w1(x)=112bx4αħ+120ax5αħ,w2(x)=112bx4αħ+120ax5αħ+112bx4αħ2 w2(x)+120ax5αħ2+1360bx6α2ħ2+1840ax7α2ħ2,w3(x)=112bx4αħ+120ax5αħ+16bx4αħ2 w3(x)+110ax5αħ2+1180bx6α2ħ2+1420ax7α2ħ2 w3(x)+112bx4αħ3+120ax5αħ3+1180bx6α2ħ3 w3(x)+1420ax7α2ħ3+bx8α3ħ320160+ax9α3ħ360480,w4(x)=112bx4αħ+120ax5αħ+14bx4αħ2 w4(x)+320ax5αħ2+1120bx6α2ħ2+1280ax7α2ħ2 w4(x)+14bx4αħ3+320ax5αħ3+160bx6α2ħ3 w4(x)+1140ax7α2ħ3+bx8α3ħ36720+ax9α3ħ320160 w4(x)+112bx4αħ4+120ax5αħ4+1120bx6α2ħ4 w4(x)+1280ax7α2ħ4+bx8α3ħ46720+ax9α3ħ420160 w4(x)+bx10α4ħ41814400+ax11α4ħ46652800,w4(x)⋮

Ten iterations are conducted and we get

(22) W10(x,ħ)=∑n=010wn(x)=w0(x) +w1(x)+w2(x)+⋯+w10(x).

By substituting (22) into the boundary conditions, we obtained four homogeneous equations. By representing the coefficient matrix of these equations with [*W*(*α*, *ξ*, *η*_1_, *η*_2_, *ħ*)] we get the following equation:

(23) $$\begin{array}{c} \left[W\left(\alpha ,\xi ,\eta_{1},\eta_{2},\hbar \right)\right]{\left[\begin{array}{cccc} a & b & c & d \end{array}\right]}^{T}={\left[\begin{array}{cccc} 0 & 0 & 0 & 0 \end{array}\right]}^{T}, \end{array}$$

where *a*, *b*, *c*, and *d* are the unknown constants of initial approximation *w*_0_(*x*) and *T* denotes the transpose of the matrix. For nontrivial solution the determinant of the coefficient matrix [*W*(*α*, *ξ*, *η*_1_, *η*_2_, *ħ*)] must vanish. Thus the problem takes the following form:

(24) $$\begin{array}{c} \text{Det}\left[W\left(\alpha ,\xi ,\eta_{1},\eta_{2},\hbar \right)\right]=0. \end{array}$$

The smallest positive real root of (24) is the critical buckling load. We defined the function *U*(*α*, *ξ*_0_, *ξ*_1_, *ζ*, *ħ*) as follows:

(25) $$\begin{array}{c} U\left(\alpha ,\xi ,\eta_{1},\eta_{2},\hbar \right)=\text{Det}\left[W\left(\alpha ,\xi ,\eta_{1},\eta_{2},\hbar \right)\right], \end{array}$$

and then we pilot the *ħ*-curves of the *U*(*α*, *ξ*, *η*_1_, *η*_2_, *ħ*) in order to find convergence region of the *ħ*.

**Figure 2:**
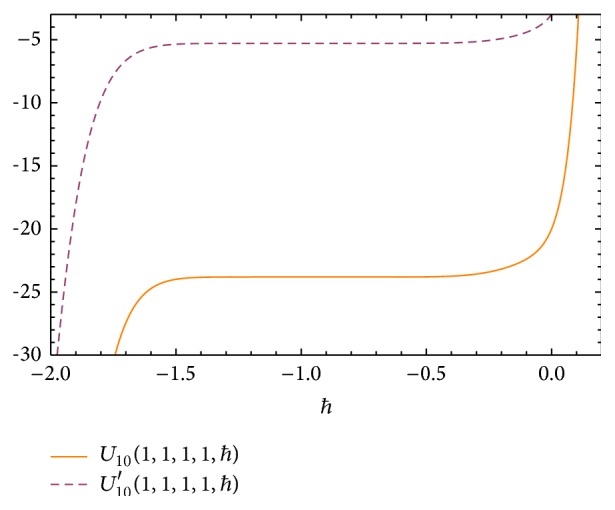
The *ħ* curves of *U*(*α*, *ξ*, *η*_1_, *η*_2_, *ħ*) and *U*′(*α*, *ξ*, *η*_1_, *η*_2_, *ħ*).

The *ħ* curves of *U*(*α*, *ξ*, *η*_1_, *η*_2_, *ħ*) and *U*′(*α*, *ξ*, *η*_1_, *η*_2_, *ħ*) are given in Figure 2. The valid region of *ħ* is the region which corresponds to the line segments nearly parallel to the horizontal axis. The valid region of *ħ* is about −1.5 < *ħ* < −0.4.

Finally we obtained the critical buckling loads from (24) for *ħ* = −0.99. We compared the exact solutions given by Wang et al. [45] and HAM solutions in Tables 2 and 3.

## 5. Conclusions

In this work, a reliable algorithm based on the HAM to solve the critical buckling load of Euler column with elastic end restraints is presented. Two cases are given to illustrate the validity and accuracy of this procedure. The series solutions of (1) by HAM contain the auxiliary parameter *ħ*. In general, by means of the so-called *ħ*-curve, it is straightforward to choose a proper value of *ħ* which ensures that the series solution is convergent. Figure 2 shows the *ħ*-curves obtained from the *m*th order HAM approximation solutions. In Tables 2 and 3 the critical buckling loads for various values of *ξ*_0_, *ξ*_1_, *ζ* obtained by HAM are tabulated. The HAM solutions and the exact solutions in [45] are compared. As a result HAM is an efficient, powerful and accurate tool for buckling loads of columns.

## Figures and Tables

**Table 1:** Stability criteria for the various columns given in Figure 1.

*ξ*	*η*_1_	*η*_2_	Stability criteria
∞	0	$-\frac{1}{1+a}$	$a\sqrt{\alpha }+\tan\sqrt{\alpha }=0$
∞	−*a*	0	$1-a\sqrt{\alpha }\tan\sqrt{\alpha }=0$

**Table 2:** Comparison of exact and HAM solutions of critical buckling loads for the column in Figure 1(a) with *η*_1_ = 0, *η*_2_ = −1/(1 + *a*), and *ξ* = ∞.

*a*	Critical load $\sqrt{\alpha }$	
	Exact solution [45]	HAM solution
0.1	2.86277	2.86277
0.2	2.65366	2.65366
0.3	2.49840	2.49840
0.4	2.38064	2.38064
0.5	2.28893	2.28893
0.6	2.21571	2.21571
0.7	2.15598	2.15598
0.8	2.10638	2.10638
0.9	2.06453	2.06453
1	2.02876	2.02876

**Table 3:** Comparison of exact and HAM solutions of critical buckling loads for the column in Figure 1(b) with *η*_1_ = −*a*, *η*_2_ = 0, and *ξ* = ∞.

*a*	Critical load $\sqrt{\alpha }$	
	Exact solution [45]	HAM solution
0.1	1.428870	1.428870
0.2	1.313840	1.313840
0.3	1.219950	1.219950
0.4	1.142230	1.142230
0.5	1.076870	1.076870
0.6	1.021110	1.021110
0.7	0.972911	0.972911
0.8	0.930757	0.930757
0.9	0.893519	0.893519
1	0.860334	0.860334
